# Vitamin D and Calcium Supplementation Reverses Tenofovir-Caused Bone Mineral Density Loss in People Taking ART or PrEP: A Systematic Review and Meta-Analysis

**DOI:** 10.3389/fnut.2022.749948

**Published:** 2022-03-31

**Authors:** Xiaoyan Bi, Fan Liu, Xiangjun Zhang, Hongyi Wang, Zehao Ye, Ke Yun, Xiaojie Huang, Haibo Ding, Wenqing Geng, Junjie Xu

**Affiliations:** ^1^National Health Commission (NHC) Key Laboratory of Acquired Immunodeficiency Syndrome (AIDS) Immunology, National Clinical Research Center for Laboratory Medicine, The First Affiliated Hospital of China Medical University, Shenyang, China; ^2^Collaborative Innovation Center for Diagnosis and Treatment of Infectious Diseases, Hangzhou, China; ^3^Key Laboratory of AIDS Immunology, Chinese Academy of Medical Sciences, Shenyang, China; ^4^Key Laboratory of AIDS Immunology of Liaoning Province, Shenyang, China; ^5^School of Public Health, The University of Tennessee, Knoxville, Knoxville, TN, United States; ^6^Department of Infectious Diseases, Beijing Youan Hospital, Capital Medical University, Beijing, China; ^7^Clinical Research Academy, Peking University Shenzhen Hospital, Peking University, Shenzhen, China

**Keywords:** tenofovir disoproxil fumarate, vitamin D, calcium, supplement, bone mineral density

## Abstract

**Background:**

The decrease of bone mineral density (BMD) after the intake of Tenofovir disoproxil fumarate (TDF)-based drugs in people living with HIV/AIDS (PLWHA) and HIV-negative key populations under pre-exposure prophylaxis (PrEP) regimen raised concerns. Previous findings on the effects of vitamin D (VD) and calcium supplements and the recovery of BMD loss were inconclusive. The optimal doses of VD and calcium and its supplementary duration remained unknown. Therefore, we conducted a systematic review and meta-analysis to synthesize current evidence on VD and calcium supplements to inform clinical practice.

**Methods:**

We searched PubMed, Web of Science, Cochrane library, and EMBASE databases for all placebo-controlled trials and prospective cohort studies published before March 5, 2021 that investigated VD and calcium supplements in participants taking TDF-based drugs. The keywords calcium, vitamin D, Tenofovir, and BMD were used for the searches. The primary outcome was changes of spine and hip BMD. A subgroup analysis was performed to determine the factors that were related to the effects of VD supplements on BMD. Locally weighted regression (loess) was used to determine the relationships of VD supplements, supplementary duration, and changes of BMD. This study was registered at PROSPERO (No. 42021231000).

**Findings:**

Seven eligible studies including 703 participants were included in the analyses. The meta-analysis found that VD and calcium supplementation was related to a significant increase of BMD in the spine and hip [standardized mean difference (SMD) 0.43; 95% CI, 0.25 to 0.61, *p* = 0.009]. Moreover, positive dose-response relationships were demonstrated between doses of VD and calcium supplements, supplementary duration, and BMD recovery. Patients who took VD with the dose level of 4,000 IU/D obtained the highest BMD improvement (SMD 0.59, 95% CI, 0.43 to 0.74). No side effects were reported on VD and calcium supplementation.

**Interpretation:**

We found the VD and calcium supplementation was associated with increases of BMD in participants taking TDF-based drugs. An optimal supplementary dose of 4,000 IU/D for VD was suggested for clinicians. The findings could be used in clinical practice to improve the BMD outcomes in people who were taking TDF-based drugs.

**Systematic Review Registration:** https://www.crd.york.ac.uk/PROSPERO/.

## Introduction

Oral antiretroviral (ART) medications have been widely applied in clinical practices to treat HIV and AIDS and to prevent HIV transmission among high-risk groups who were taking pre-exposure prophylaxis (PrEP). Medications that contain TDF (tenofovir disoproxil fumarate) were the most commonly used ART medications for people living with HIV/AIDS (PLWHA) and accounted for ~63.1% of ART medication use (1, 2). Moreover, TDF-based drugs could be used as a single-medication regimen for PrEP prevention, and it was recommended by US Centers for Disease Control and Prevention (CDC) and the WHO (3–5). However, studies reported that people who took TDF drugs have had the side effect of bone density decrease (6). The bone loss fluctuated between 2 and 6% yearly during the first year of ART initiation (7–9), and more severe outcomes included osteoporosis and fragility fractures (10, 11). Studies found that bone mineral density (BMD) loss was associated with PrEP compliance (12–14); therefore, this side effect became a barrier for PrEP receipt. One study reported that 42.9% of men who have sex with men were not willing to use PrEP because of the fear of reduced BMD (15).

**Figure 1:**
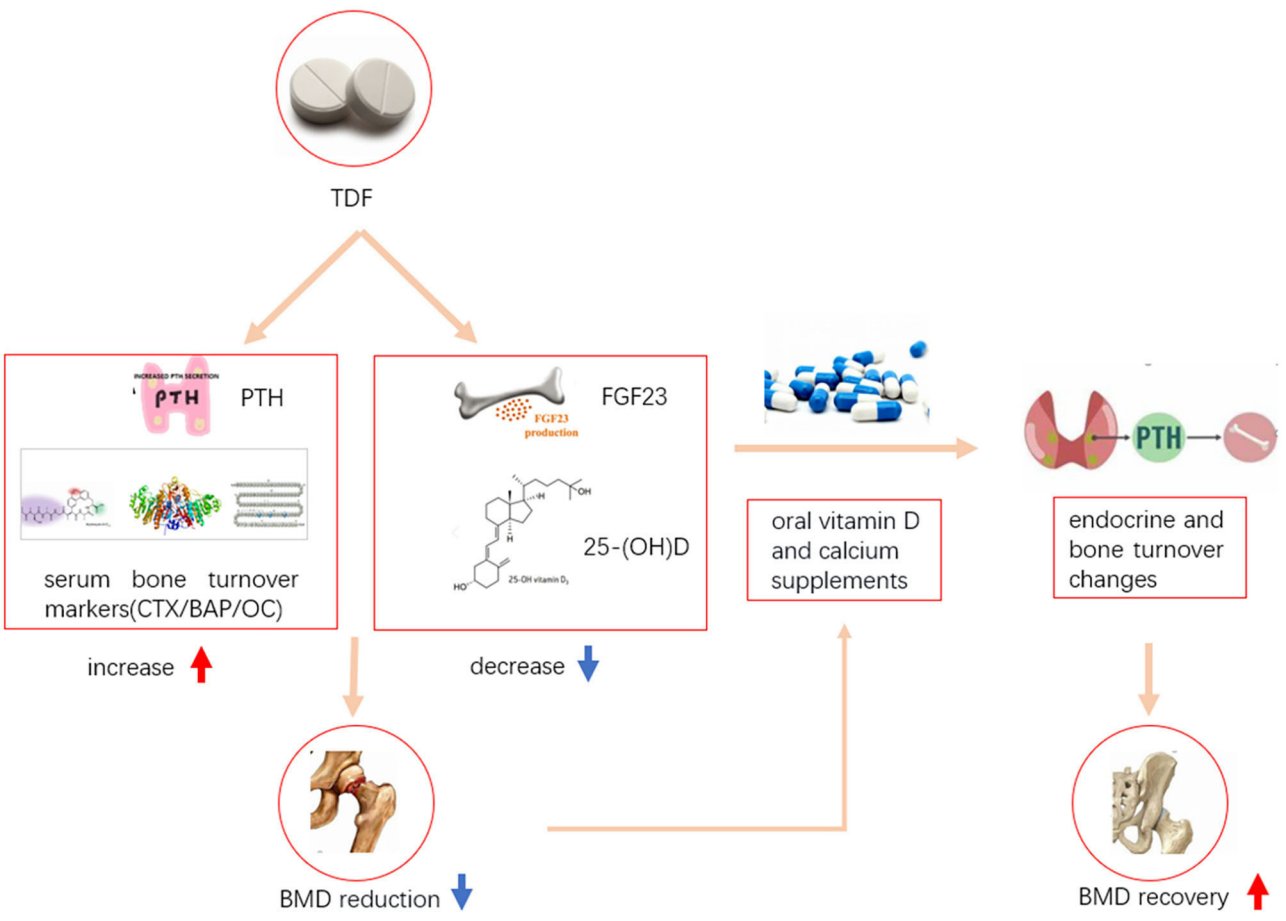
Possible mechanisms of how TDF caused BMD reduction and how oral vitamin D and calcium supplementation alleviated BMD reduction. TDF, Tenofovir disoproxil fumarate; PTH, parathyroid hormone; FGF23, fibroblast growth factor 23; CTX, C-terminal telopeptides; BAP, bone alkaline phosphatase; OC, osteocalcin; 25-(OH)D, 25-hydroxy vitamin D; BMD, bone mineral density.

Although the mechanisms of how TDF could have caused BMD loss have not been fully explained in the literature, we underlined some possible pathways to indicate this process based on current evidence. TDF could increase the level of parathyroid hormone (PTH), decrease the level of fibroblast growth factor 23 (FGF23), and affect the levels of some serum bone turnover markers [such as, C-terminal telopeptides (CTX), bone alkaline phosphatase (BAP), osteocalcin (OC), and 25-hydroxy vitamin D (25-OHD)](16, 17), which in turn caused BMD loss. Oral vitamin D (VD) and calcium supplementation could alleviate this effect on BMD through changing the levels of endocrine and bone turnover markers (12, 18) (Figure 1).

Some placebo-controlled, randomized controlled trials (RCTs) and observational studies have investigated the effects of VD and calcium supplementation on the recovery of bone loss (19–25). However, literature did not have conclusive results without meta-analysis and systematic review studies' support. Also, no dose-response relationship has been reported. One study found that VD3 plus calcium supplements mitigates the BMD loss in HIV-positive populations who initiated TDF (25). Another study found similar results in HIV-negative populations taking PrEP (21). However, other studies did not find significant differences in changes of the hip and lumbar bone density among people living with HIV/AIDS (PLWHA) in both the VD and calcium supplementation group and the placebo group (26). Another study reported that there was no difference in the lumbar spine BMD in HIV-negative participants who participated in PrEP studies (−2.11 vs. −2.14%, *p* = 0.40) (27). The sample sizes were small and these studies were conducted in small regions, so that the generalizability of these findings was limited. Furthermore, the optimal amount of VD and calcium and the duration of supplementation remained unknown.

Therefore, to fill in the knowledge gaps, a systematic review and meta-analysis were performed to evaluate the effects of VD and calcium supplementation and factors associated with BMD recovery among people who were taking TDF-based drug. In addition, we investigated the dose-response relationships among the dose of VD and calcium supplements, length of supplementation, and BMD changes. The findings could be used to guide clinical practices for TDF-drug prescription and management of the TDF-drug-related BMD loss in both PLWHA and HIV-negative people who take PrEP.

## Methods

### Search Strategies and Selection Criteria

This systematic review was registered at the PROSPERO (International Prospective Register of Systematic Reviews) (No. 42021231000) (28).

We searched PubMed, Web of Science, Cochrane library, and EMBASE databases from the inception date to March 5, 2021, using the keywords calcium, vitamin D, Tenofovir, and bone mineral density (BMD), without language restrictions. We searched studies that investigated VD and calcium supplements and BMD in PLWHA who took TDF or HIV-negative population who took Tenofovir/Emtricitabine for PrEP. We used the index term (((‘Vitamin D') OR (‘vitamin-D') OR (VitD) OR (‘Vit-D') OR (VD)) OR ((Calcium) OR (Ca))) AND (((Tenofovir) OR (‘Tenofovir disoproxil fumarate') OR (‘tenofovir disoproxil') OR (TDF)) OR ((‘Antiretroviral Therapy') OR (‘antiretroviral therapy') OR (ART) OR (HAART) OR (‘antiretroviral treatment') OR (‘Antiretroviral-Treated'))) AND (((‘Bone Density') OR (‘bone mineral density') OR (BMD) OR (‘bone density') OR (Osteoporosis) OR (‘bone loss') OR (‘bone rarefaction') OR (‘skeletal rarefaction') OR (OP) OR (‘rarefaction of bone') OR (‘Bone fractures') OR (fracture))) for the search. We also included eligible studies through screening of the references of relevant studies and reviews. Moreover, we contacted authors to obtain articles that were not available online.

Studies were selected based on the following inclusion criteria: (1) participants were patients who are HIV-1 positive or key populations who took PrEP drugs; (2)participants used TDF-based drugs; (3) studies investigated VD or calcium supplements; (3) studies reported the outcomes of BMD for a *z*-score or a t-score; (4) studies had a control group. Exclusion criteria were (1) studies used indirect measures [e.g., 25 (OH) D)]; (2) studies reported the outcomes by absolute values instead of changes of BMD; (3) the detection site was neither spine nor hip. All study titles and abstracts were screened by two reviewers (XYB and FL) independently. Disagreements were resolved through consensus.

Commonly, VD and calcium were given orally and BMD was measured by a dual-energy X-ray absorptiometry (DXA General Electric Healthcare, Madison, WI, USA) scan. A DXA scan was generally read in a blinded fashion using a standardized protocol (29). A clinically significant change was defined as a decline of ≥3.0% for the lumbar spine and ≥4% for the femoral neck and total hip (27). Adverse effects associated with VD supplementation were also collected for the selected studies.

### Data Analysis

Two reviewers extracted the following data independently from the 9 eligible studies: lead author, year of publication, intervention and control regimens, VD dosing, changes of BMD, and study duration. The participants' baseline characteristics were also collected including age, sex, continent of origin, and ART drugs. Disagreements were resolved by consensus.

The 9 studies were assessed using measures and criteria at the Cochrane Handbook for Interventions for quality control (18). Measures that were used to evaluate the study quality included randomization sequence generation, allocation concealment, blinding of participants and research personnel, blinding of outcome assessment, outcome data completion, selectivity of reporting, and other measures that could possibly lead to biases. All eligible studies were rated as high quality (A), moderate quality (B), or low quality (C). In addition, the Newcastle-Ottawa Scale (NOS) was used to evaluate all non-RCT studies (30). Measures that were used to evaluate biases in each study included selection, comparability, and exposure using a score ranging from 0 to 10. Discrepancies were resolved by consensus of two reviewers.

The changes of spine and hip BMD were the primary outcome. Previous studies concluded that there was a 2 to 6% hip and spine BMD loss at the first 24 to 48 weeks of ART initiation (7, 31, 32), which potentially was caused by TDF-based drugs (33).

Statistical heterogeneity between studies was assessed using an I2 scoring system. An I2 score of >50% was considered significant heterogeneity. The units we included were different, so we calculated the absolute risks by multiplying SMD and its 95% CI with the baseline risk. To assess the possible modification effect of clinical characteristics on the relationship of VD supplements and BMD, subgroup analyses were performed, including levels of detection site, HIV infection status, continent of origin, doses of VD and calcium, study duration, age, and gender by random effects. A sensitivity analysis was performed for studies with high- and moderate- quality. In addition, studies that recruited participants under specific conditions or tasks were excluded from the sensitivity analysis. The Egger's test was used to assess possible publication bias.

All meta-analyses were performed using the Stata 16 and Review Manager (5.3) for forest map and sub-group analysis. Additionally, we used the SAS (9.4) for the locally weighted regression (loess) method to assess the dose-response relationships between VD and calcium supplementation, study duration, and changes of BMD. All tests were 2-tailed with a statistical significance level of *p* < 0.05.

## Results

### Study Retrieval and Selection

**Figure 2:**
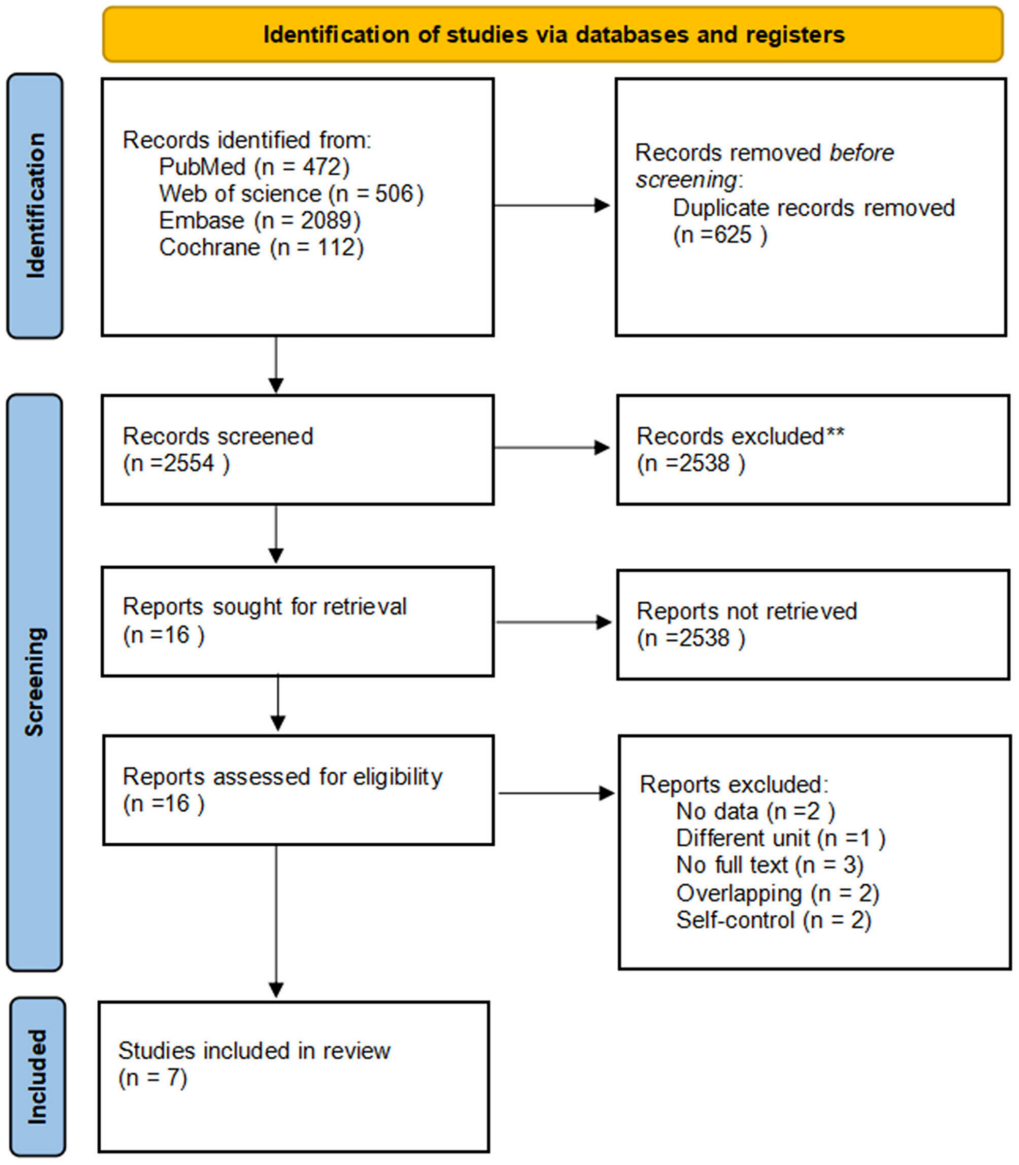
Literature search and study selection process. ** means the records irrelevant to the subject (n = 2538).

Using the search strategies, we identified 3,179 studies. After the removal of duplicates, 2,554 unique records were screened, and 16 full-text were assessed for eligibility. We excluded 9 studies for the following reasons: duplicate publications (2), no available BMD data (1), different unit was used (1), self-controlled studies (2), full text unavailability (3). Seven studies were identified. The full texts of the seven articles were carefully reviewed, and all of them met the inclusion criteria. Finally, we included seven studies in the analyses (Figure 2). A summary of the seven studies was included in Table 1.

**Table 1:** Characteristics of the included trials and participants.

	**Included studies**	**Continent**	***N* (Vitamin D vs. placebo or no treatment)**	**Gender (male vs. female)**	**Age, y (IQR or SD)^**a**^**	**HIV infection status**	**VD oral intake dosage/ frequency**	**Ca oral intake dosage**	**Treatment duration**	**Change of spine BMD (%)^**c**^ (vitamin D vs. placebo or no treatment)**	**Change of hip BMD (%) (Vitamin D vs. placebo or no treatment)**	**Quality evaluation**
1	Havens et al. (24)	America	99 vs. 89	179 vs. 33	22.0 (21.0–23.0)	HIV-positive	50,000 IU/4 W	0	48 W	1.15% (−0.75 to 2.74%) vs. 0.09% (−1.49 to 2.61%)	−0.17% (−2.12 to 1.73%) vs. −0.42% (−1.66 to 0.71%)	A
2	Overton et al. (25)	America	79 vs. 86	149 vs. 16	36 (28–47)	HIV-positive	4,000 IU/D	1,000 mg	48 W	−1.23% (−3.73 to 0.20%) vs. −2.94% (−4.87 to −0.94%)	−1.36% (−3.43 to 0.50%) vs. −3.22% (−5.56 to −0.88%)	A
3	Boontanondha et al. (26)	Thailand	9 vs. 9	17 vs. 1	30.3 (±8.9)	HIV-positive	20,000 IU/W	1,250 mg	24 W	−3.6% (−4.4 to −2.5%) vs. −4% (−4.9 to −1.3%)	−2.7% (−4.9 to −1.6%) vs. −2.8% (−4.3 to −2.5%)	B
4	Pornpaisalsakul et al. (21)	Thailand	38 vs. 42	66 vs. 14	18 (17–20)	HIV-negative	400 IU/D	1,200 mg	24 W	0.05% (0–0.05%) vs. 0.03% (−0.1 to 0.03%)	NA^b^	B
5	Nct (22)	America	79 vs. 86	149 vs. 16	18–65	HIV-positive	4,000 IU/D	1,000 mg	48 W	−1.41% (−3.78–0.00%) vs. −2.91% (−4.84 to −1.06%)	−1.46% (−3.16 to −0.40%) vs. −3.19% (−5.12 to −1.02%)	A
6	Puthanakit et al. (20)	Thailand	24 vs. 24	NA (49%)	14.3 (13.0–15.5)	HIV-negative	400 IU/D	1,500 mg	6 M	0.65 (0.13–1.20) vs. −0.50 (−1.00%to−0.06)	NA	B
7	Kortenaar et al. (27)	Canada	24 vs. 15	39 vs. 0	34 (29–40)	HIV-negative	1,000 IU/D	0–1,000 mg	12 M	−2.11% (−2.61 to 1.40%) vs. −2.14% (−4.01 to −1.45%)	−0.89% (−2.76 to −0.09%) vs. −0.69% (−2.23 to 1.58%)	A

*^a^Stands for mean age (±SD) for 30.3 (±8.9); stands for all for 18–65, stands for IQR (interquartile range) for others*.

*^b^Not available*.

*^c^Change of spine/hip BMD (bone mineral density) means the percentage increase*.

The seven studies (13–15, 17–20) were conducted in three countries and enrolled 703 participants (median 118, range 18–188, IQR 39–165). Studies included men (86.9%) and women participants who aged from 13 to 65 years old. Five studies (13–15, 17–19) were intervention trials, and one (20) was prospective cohort study. We included participants who were HIV positive and treated using TDF (California, USA) (*N* = 5 36) and HIV-negative participants who were under Tenofovir/Emtricitabine PrEP (*N* = 167), and all of them were supplemented with VD (Tishcon, Westbury, New York or Charoon Bhesaj, Thailand; ranging from 400 to 4,000 IU/D) and calcium (ranging from 0 to 1,500 mg/D). The median follow-up duration across studies was 48 weeks (ranging from 24 to 48 weeks). All studies detected changes of spine BMD, and five of them (15, 17–20) reported changes in hip BMD. All studies were determined as a low-risk level of biases (Supplementary Table 1).

### Meta-Analysis

**Figure 3:**
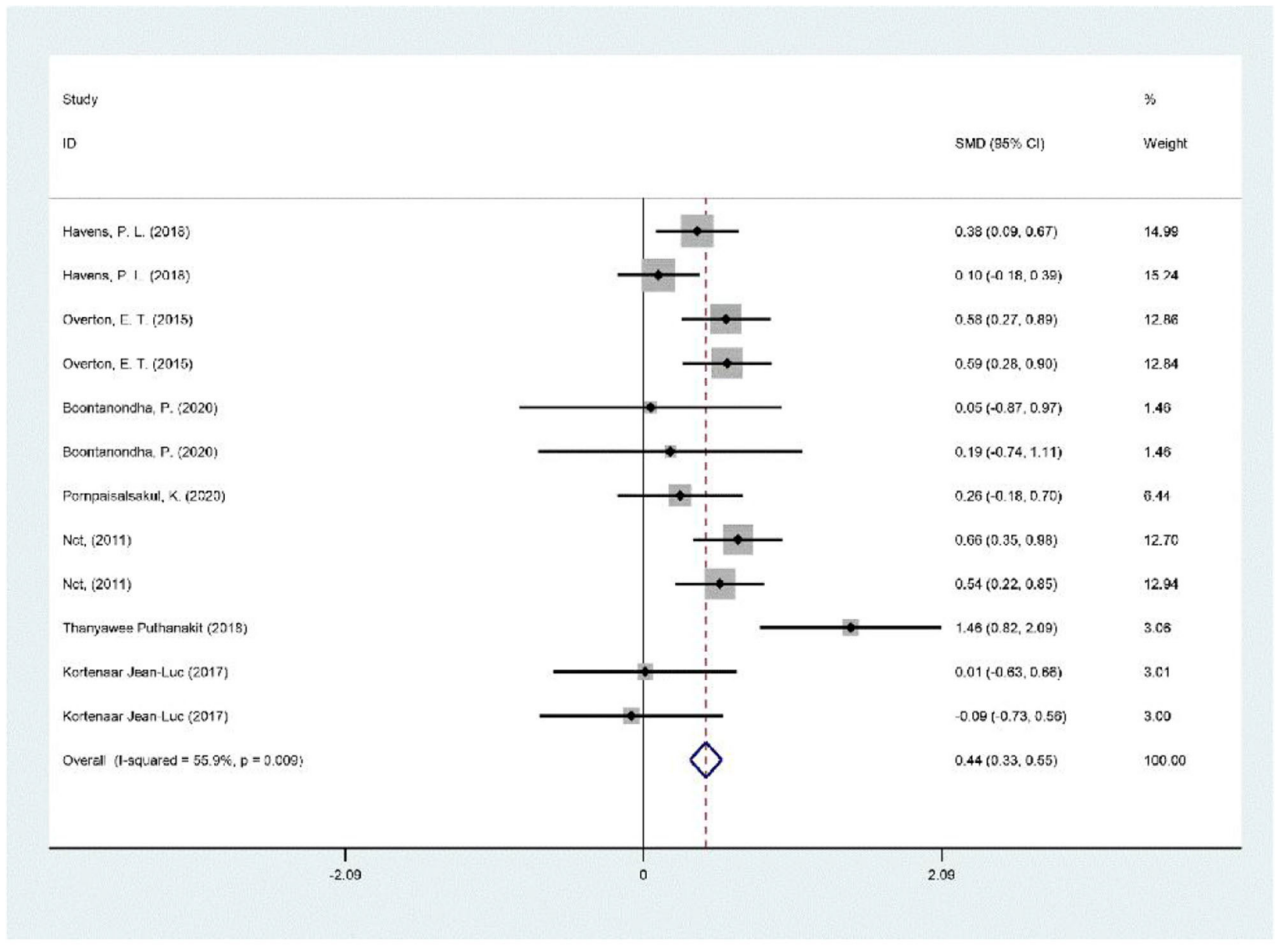
Meta-analysis results of vitamin D supplementation on BMD changes.

Each of the seven studies had a VD and calcium supplementation group and a placebo or non-treatment group. There was a significant positive correlation between VD supplementation and BMD increase, as shown in Figure 3 (SMD 0.43, 95% CI 0.25 to 0.61, *p* = 0.009).

### Subgroup Analysis

**Figure 4:**
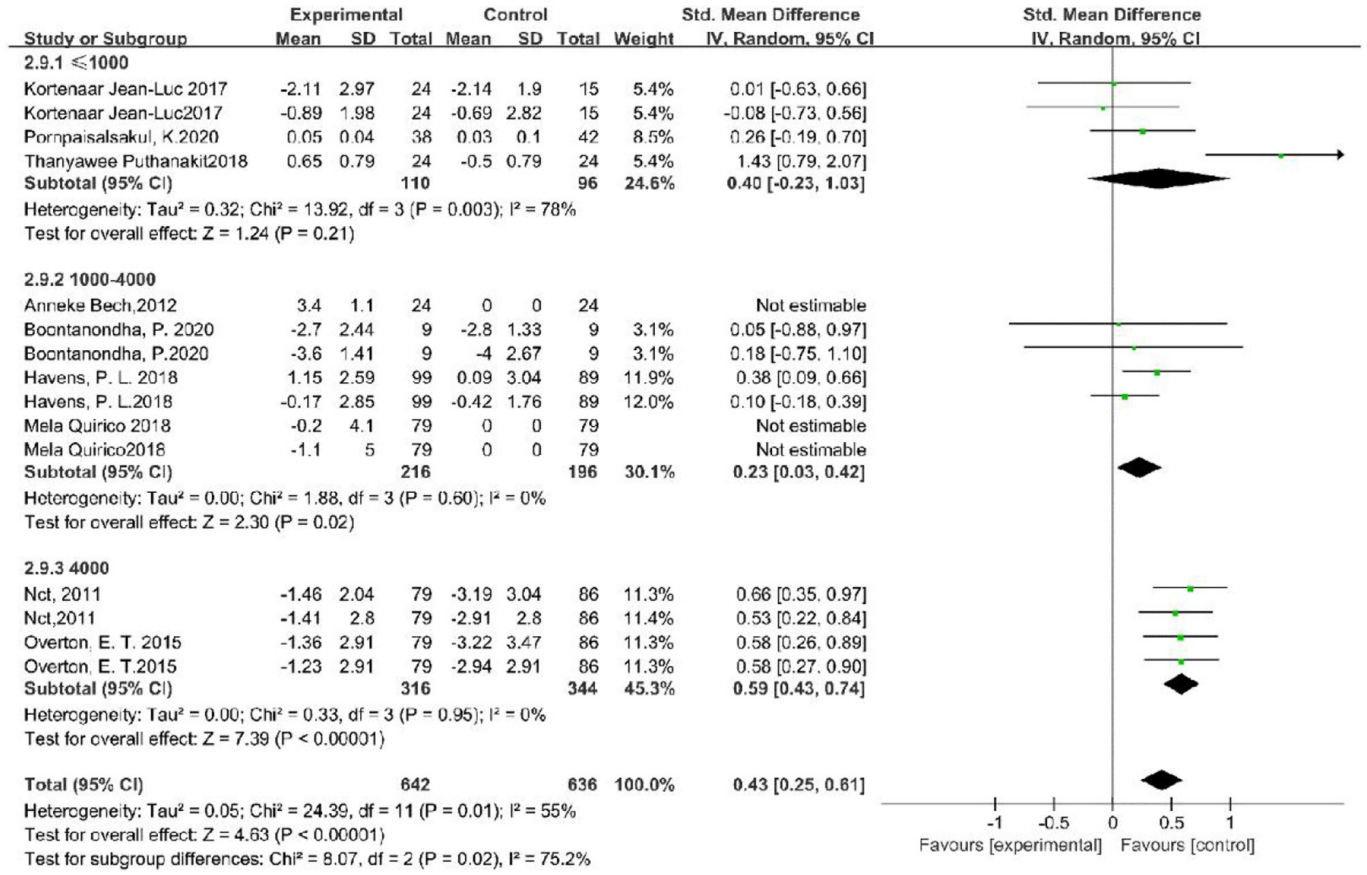
Subgroup analysis of the association between vitamin D supplementation dosing and BMD increase.

Substantial heterogeneity (*I*^2^ = 55%) was used as a functional outcome. We conducted multiple subgroups and sensitivity analyses to examine the effects of oral VD and calcium supplementation on BMD changes by participants' continent, age, sex, site of detection, and study-related factors. Functional outcomes were significantly better among people whose VD supplement was 4,000 IU/D (SMD, 0.30; 95% CI, 0.02–0.58, *p* = 0.006). In subgroup analyses, there were no differences in treatment outcomes regarding age (*p* = 0.42), sex (*p* = 0.48), HIV infection status (*p* = 0.89), continent (*p* = 0.1), and site of detection (*p* = 0.45, Table 2 and Figure 4).

**Table 2:** Subgroup analysis of association between vitamin D supplementation and BMD changes.

**Variable**	**No. of records**	**No. of participants**	**I-squared (%)**	**SMD (95% CI)**	***P*-value**	**P for interaction**
**Age (years)**
<30	4	504	79	0.46 [0.05, 0.88]	0.003	0.42
30–40	6	444	26	0.38 [0.13, 0.62]	0.24	
>40	2	330	0	0.60 [0.38, 0.82]	0.57	
**Proportion of male**
>70%	10	1,150	37	0.40 [0.24, 0.55]	0.11	0.48
<70%	2	128	89	0.82 [−0.33, 1.97]	0.003	
**Study continent**
North America	6	784	53	0.33 [0.11, 0.56]	0.06	0.1
Asia	3	116	0	0.21 [−0.15, 0.58]	0.92	
Europe	3	378	67	0.77 [0.37, 1.16]	0.05	
**HIV infection status**
HIV-1 positive	8	1,072	32	0.45 [0.29, 0.60]	0.17	0.89
HIV-1 negative (PrEP)	4	206	78	0.40 [−0.23, 1.03]	0.003	
**Site of detection**
Spine	7	703	54	0.49 [0.24, 0.73]	0.04	0.45
Hip	5	575	62	0.34 [0.04, 0.64]	0.03	
**Vitamin D intake dosage**
4,000 IU/D	4	660	0	0.59 [0.43, 0.74]	0.003	0.02
1,000–4,000 IU/D	4	412	0	0.23 [0.03, 0.42]	0.6	
≤ 1,000 IU/D	4	206	78	0.40 [−0.23, 1.03]	0.95	
**Vitamin D supplement duration**
24 weeks	4	164	72	0.51 [−0.15, 1.17]	0.01	0.77
48 weeks	8	1,114	48	0.41 [0.24, 0.58]	0.06	
**Calcium supplement dosage**
≤ 1,000 mg/D	8	1,114	48	0.41 [0.24, 0.58]	0.06	0.77
>1,000 mg/D	4	164	72	0.51 [−0.15, 1.17]	0.01	

*SMD, standardized mean difference; PrEP, pre-exposure prophylaxis*.

### Dose-Response Relationship

**Figure 5:**
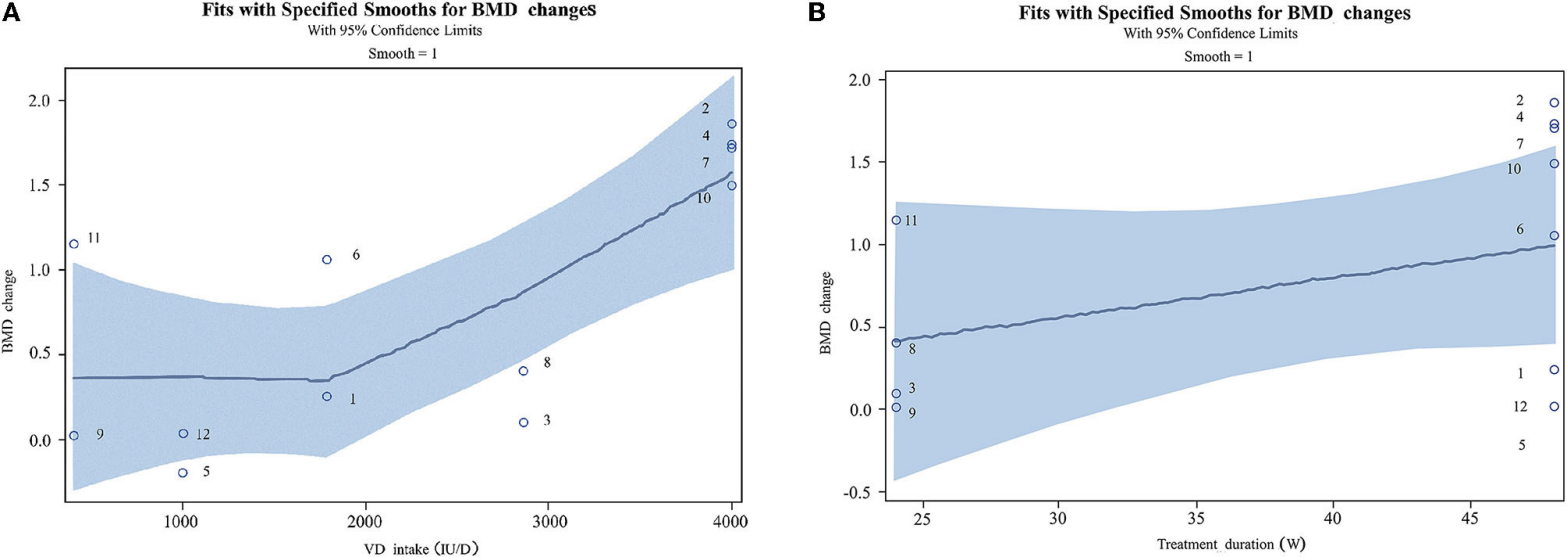
Correlations among vitamin D supplement, treatment duration, and the changes of BMD (The corresponding articles were included in Supplementary Table 2). **(A)** Dose-response relationship between vitamin D and calcium supplementation and BMD changes. **(B)** Dose-response relationship between treatment duration and BMD changes.

This study found positive correlations between VD and calcium supplementation, study duration, and BMD increases (Figure 5). Participants' BMD showed a gradual increase with the increase of VD supplementation at the range of 50,000 IU/4 W to 4,000 IU. A VD3 daily dosing of 4,000 IU was identified as the optimal dose. There was a positive correlation between supplementary the time and the increase of BMD within 48 weeks.

### Sensitivity Analysis and Publication Bias

Sensitivity analyses suggested that the effect size of the studies was within the 95% confidence interval (Supplementary Figure 1), which indicated that the study results were stable. The heterogeneity score for this meta-analysis was 55%, which implied a moderate heterogeneity. The funnel plot and the Egger's asymmetry test were used to evaluate the publication bias. The Egger's asymmetry test results showed no evidence of publication bias (*p* = 0.700, Supplementary Figure 2).

## Discussion

This study concluded that VD supplement was significantly associated with the recovery of BMD in people who took TDF-based drugs through meta-analysis. We also found a dose-response relationship between VD supplement dosage, supplementary length, and the recovery of BMD, and the optimal dosage of VD supplement was 4 000 IU cholecalciferol (VD3) per day. BMD continuously increased up to 48 weeks of VD supplement. These findings filled in the knowledge gaps and provided useful guidance for HIV clinical doctors to treat BMD loss in participants who use TDF-based regimens. The findings in this study may help to remove concerns about potential PrEP side effects on BMD, therefore improving the receipt of PrEP.

This study indicated that compared with non-supplementation group, the BMD in people who used VD could be improved to a normal level, which was a pooled SMD of 0.43. Some clinicians recommended to prescribe upgraded ART drugs to eliminate the potential risks (34–36). Also the updated guidelines for PrEP in the United States recommended the use of updated PrEP drugs (emtricitabine/tenofovir alafenamide [Descovy^®^ F/TAF]) (37) because of its less side effect on bone and good efficacy (38). However, these updated drugs were more expensive. The price of TDF in China was ¥1,980 RMB/bottle (30 tablets), while that of TAF was ¥2,280 RMB (30 tablets). People must spend ¥3,600 RMB more for one-year intake of TAF. Moreover, the price of VD was ¥44.7 RMB/bottle (30 tablets) and ¥536.4 for one-year intake. The TAF regimen was 6.7 times of TDF plus VD solution. Therefore, our study indicated that VD supplementation could provide a cost-saving solution for people who had a long-term use of TDF drugs, especially for people in developing countries and regions with limited resources. Also, it was suggested to add the supplementation of VD and calcium into clinical guidelines to improve treatment outcomes.

The subgroup analysis and dose-response relationship analysis demonstrated that some factors were associated with VD supplement and BMD recovery in people who took TDF drugs. First, we found that people who used high-dose and long-time VD had better BMD recovery. Additionally, we found that there was a dose-response relationship between dose, supplement duration, and BMD changes for the range of 0–4,000 IU VD supplement per day within 48 weeks. It meant that taking 4,000 IU per day for 48 weeks could help increase the BMD continuously. Moreover, this study did not find drug toxicity and side effects in people who took high VD doses and used VD for longer time. In addition, we found that the results did not differ in terms of age, gender, country, HIV infection status, and site of detection. There were two possible reasons. First, these factors were not associated with BMD. Second, possible relationships were not detected due to the small sample size and short study duration of these studies. Therefore, future meta-analysis studies could re-evaluate the relationships by including more participants and long-duration studies.

The study had strengths. This systematic review was conducted following a prespecified and registered protocol using Cochrane methodology, which ensured a good validity. Also, we performed a comprehensive search with including multiple databases and clinical trial registries. The search did not restrict on languages and publication status of the trials. Likely, we included all available literature on this topic. In addition, to our knowledge, this was the first study that concluded on relationships among BMD changes over time, VD dosing, and the optimal VD dose and prescription time.

The study had limitations. First, a few studies that used intermediate indicators such as parathyroid hormone (PTH) and procollagen I N-terminal peptide (PINP) as measures of BMD were excluded. Bias could possibly occur due to the exclusion of these studies. Second, the studies that were included in the current meta-analysis had small sample sizes. Each study included <250 participants with a total of 703 participants for this meta-analysis. However, this study was able to detect significant relationships among variables although the sample size was small.

In conclusion, our meta-analysis synthesized findings of current studies about the effects of VD and calcium supplementation on the recovery of BMD and indicated that VD supplementation improved BMD. The study provided valuable implications for clinical doctors to prescribe VD for people who took TDF-based drugs to reverse its effect of BMD loss. A daily supplement dosing of 4,000 IU of VD that could achieve the optimal outcome was recommended. Moreover, it was a cost-saving method and could be applied in HIV treatment facilities and PrEP prevention programs especially in resource-limited settings.

## Data Availability Statement

The original contributions presented in the study are included in the article/Supplementary Material, further inquiries can be directed to the corresponding author/s.

## Author Contributions

XB and JX conceived and designed the study, analyzed the data, and interpreted results. XB and FL acquired the data, screened records, extracted data, and assessed risks of biases. XB designed the literature search and conducted the statistical analyses. XB, FL, JX, XZ, HW, XH, KY, ZY, WG, and HD wrote, revised, and interpreted the study results of the manuscript. All authors have read and approved the final manuscript before the submission.

## Funding

This work was supported by the National Natural Science Foundation of China (81872674), the Mega-Projects of National Science Research [13th Five-Year Plan (2017ZX10201101-002-007)], the Beijing Excellent Talent Plan (2018000021223ZK04), and National Science and Technology Major Project (2018ZX10101).

## Conflict of Interest

The authors declare that the research was conducted in the absence of any commercial or financial relationships that could be construed as a potential conflict of interest.

## Publisher's Note

All claims expressed in this article are solely those of the authors and do not necessarily represent those of their affiliated organizations, or those of the publisher, the editors and the reviewers. Any product that may be evaluated in this article, or claim that may be made by its manufacturer, is not guaranteed or endorsed by the publisher.
