# Supplementary material for: Vitamin D and Calcium Supplementation Reverses Tenofovir-Caused Bone Mineral Density Loss in People Taking ART or PrEP: A Systematic Review and Meta-Analysis
Source: Front Nutr. 2022 Mar 31;9:749948. doi: 10.3389/fnut.2022.749948 (PMC9008884; doi:10.3389/fnut.2022.749948)
Supplement: Supplementary file 1 [file Data_Sheet_1.docx]

Supplementary Material

# Supplementary Figures and Tables

## Supplementary Figures
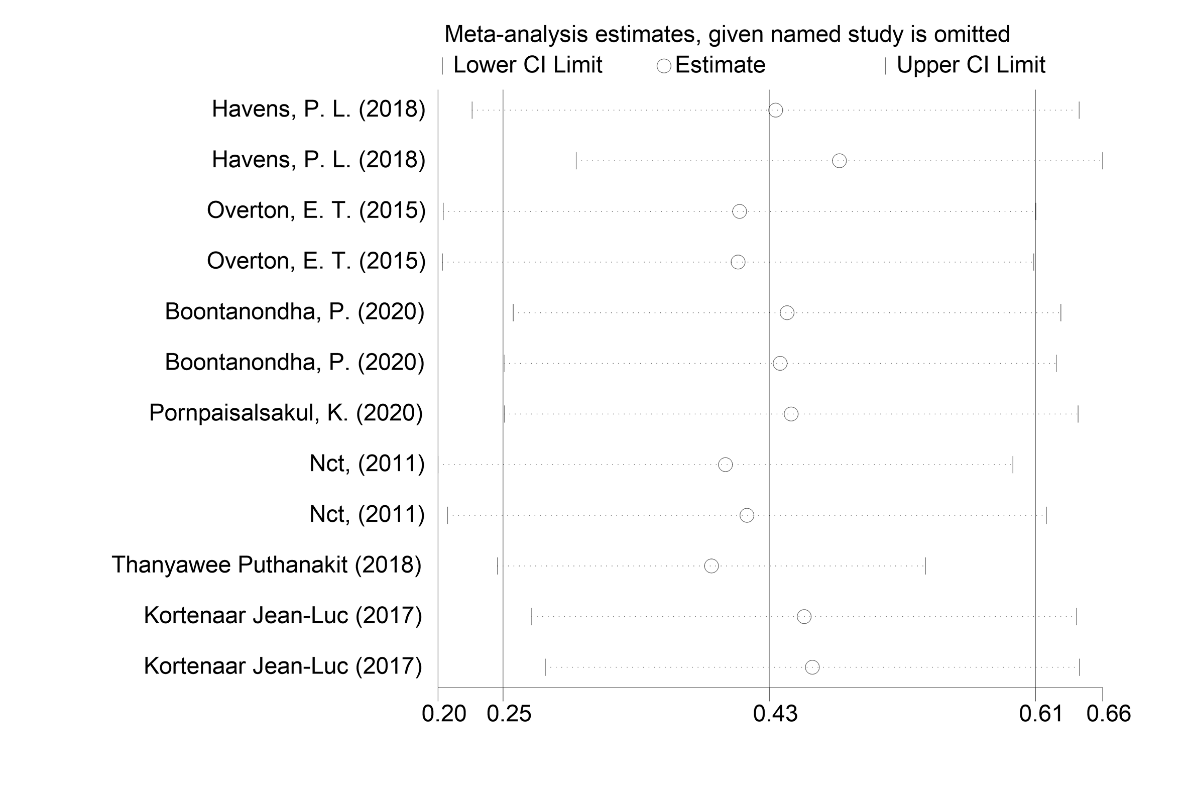
Supplementary Figure 1. Sensitivity analysis for seven studies.


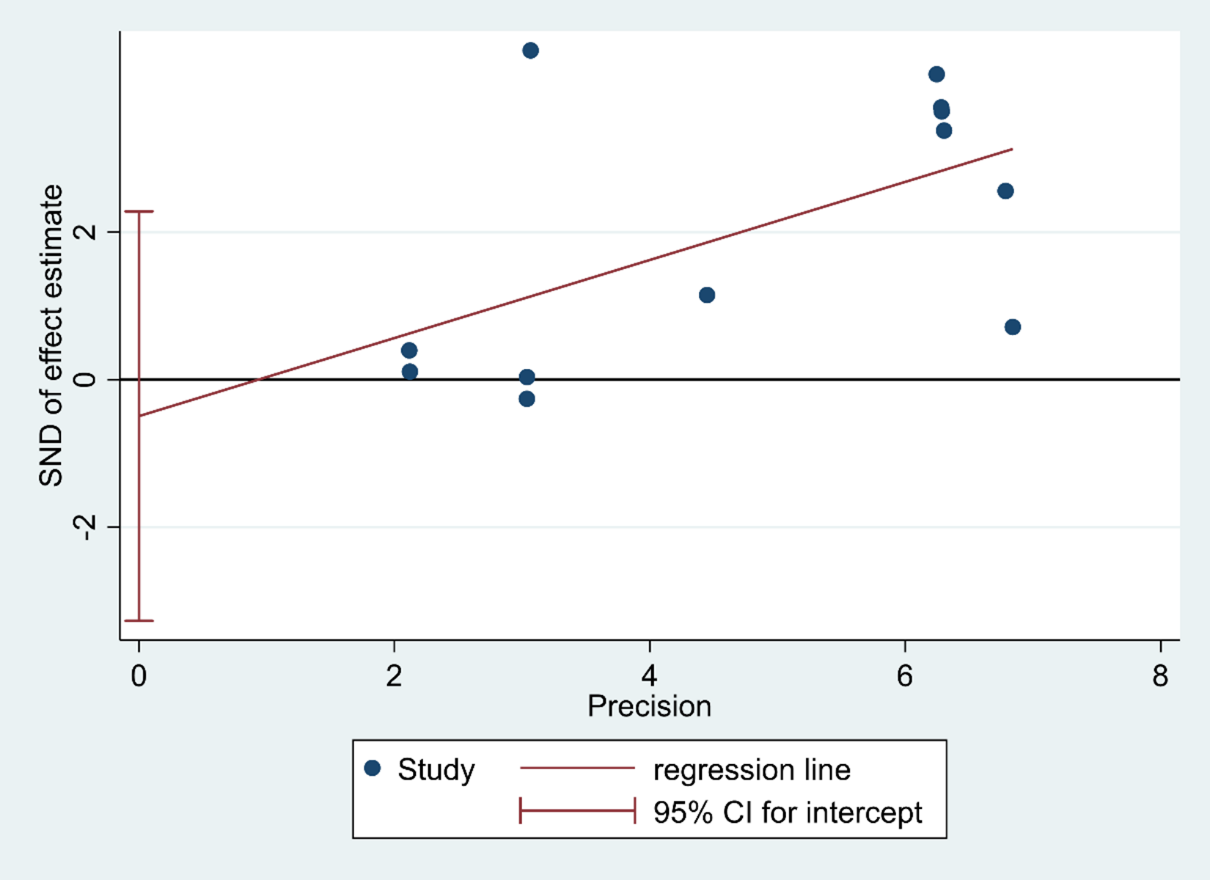


**Supplementary Figure 2.** Egger’s asymmetry test for seven studies.

## Supplementary Tables

**Appendix Table 1.** Quality evaluation of seven studies

| Literature | Item | | Authors' judgement | | Description | | Study gradings |
| --- | --- | --- | --- | --- | --- | --- | --- |
| Vitamin D3 Supplementation Increases Spine Bone Mineral Density in Adolescents and Young Adults with Human Immunodeficiency Virus Infection Being Treated With Tenofovir Disoproxil Fumarate: A Randomized, Placebo-Controlled Trial. | adequate sequence generation | | | unclear | | insufficient information about the sequence generation process to permit judgement of 'yes' or 'no' | A |
|  | allocation concealment | | | yes | | Participants were randomized to the treatment group of receiving vitamin D3, 50 000 IU  (Tishcon, Westbury, New York) or a matching placebo gelatin capsule |  |
|  | blinding | | | yes | | Treatment assignment was blinded to all study participants and personnel except the site pharmacist. |  |
|  | incomplete outcome data addressed | | | yes | | Drop out recorded was excluded in analysis. |  |
|  | free of other bias | | | yes | | The study was free of other sources of bias. |  |
| Vitamin D and Calcium Attenuate Bone Loss with Antiretroviral Therapy Initiation: A Randomized Trial. | adequate sequence generation | | | yes | | Randomization was done using Web-based access to a central computer system maintained by the Frontier Science & Technology Research Foundation (Buffalo, New York). | A |
|  | allocation concealment | | | yes | | identically matching placebos (Tishcon) |  |
|  | blinding | | | yes | | At the time of discovery and while blinded to treatment group assignment and all on-study data |  |
|  | incomplete outcome data addressed | | | yes | | the protocol team decided that these patients could continue to receive study treatment but their data would not be included in efficacy analyses |  |
|  | free of other bias | | | unclear | | study was done in the United States and Puerto Rico, and 90% of the patients were men; thus, generalizability to other areas and women may be limited |  |
| Vitamin D and Calcium Supplement Attenuate Bone Loss among HIV Infected Patients Receiving Tenofovir Disoproxil Fumarate/Emtricitabine/ Efavirenz: An Open-Label, Randomized Controlled Trial. | adequate sequence generation | | | unclear | | insufficient information about the sequence generation process to permit judgement of 'yes' or 'no' | B |
|  | allocation concealment | | | unclear | | taken with food |  |
|  | blinding | | | unclear | | The study did not address this outcome. |  |
|  | incomplete outcome data addressed | | | yes | | All patients completed study follow-up |  |
|  | free of other bias | | | unclear | | Most study patients were male and men who have sex with men; thus, the results may be difficult to generalize to other populations, such as females and HIV-infected individuals with heterosexual risk. |  |
| Effects of vitamin D and calcium supplementation on bone mineral density among Thai youth using daily HIV pre-exposure prophylaxis. | adequate sequence generation | | | yes | | Study participants were allocated by blocks of four random inaction with the concealment method using an opaque sealed envelope technique | B |
|  | allocation concealment | | | unclear | | vitamin D and calcium supplementation with meals twice daily |  |
|  | blinding | | | unclear | | The study did not address this outcome. |  |
|  | incomplete outcome data addressed | | | yes | | 21 participants in this study were lost to follow-up, 13 participants in arm A and eight participants in arm B, there were no differences seen between arms |  |
|  | free of other bias | | | unclear | | the absence of a placebo control arm in this study ultimately means that apparent benefits seen cannot be fully ascribed to vitamin D and calcium supplementation |  |
| High Dose Vitamin D and Calcium for Bone Health in Individuals Initiating HAART | adequate sequence generation | | | unclear | | The study did not address this outcome. | A |
|  | allocation concealment | | | yes | | A placebo for vitamin D3 once daily taken orally as one capsule with food for 48 weeks. |  |
|  | blinding | | | yes | | Double (Participant, Investigator) |  |
|  | incomplete outcome data addressed | | | yes | | Includes only subjects who did not have eligibility violations. |  |
|  | free of other bias | | | yes | | The study appears to be free of other sources of bias. |  |
| Effect of calcium and vitamin D supplementation on bone mineral accrual among HIV-infected Thai adolescents with low bone mineral density | adequate sequence generation | | | no | | Self-control | C |
|  | allocation concealment | | | no | | Self-control |  |
|  | blinding | | | no | | Self-control |  |
|  | incomplete outcome data addressed | | | yes | | two were lost to follow-up (both had low BMD with VDD) |  |
|  | free of other bias | | | yes | | The study appears to be free of other sources of bias. |  |
| Bone mass preservation with high-dose cholecalciferol and dietary calcium in HIV patients following antiretroviral therapy. Is it possible? | adequate sequence generation | | | no | | Self-control | C |
|  | allocation concealment | | | no | | Self-control |  |
|  | blinding | | | no | | Self-control |  |
|  | incomplete outcome data addressed | | | yes | | All patients underwent baseline (T0) and follow-up (T1) DXA evaluation focused on the spine (L1–L4) and total hip |  |
|  | free of other bias | | | yes | | The study appears to be free of other sources of bias. |  |
| Treatment of Calcium and Vitamin D Deficiency in HIV-Positive Men on Tenofovir-Containing Antiretroviral Therapy | adequate sequence generation | | | no | | Self-control | C |
|  | allocation concealment | | | no | | Self-control |  |
|  | blinding | | | no | | Self-control |  |
|  | incomplete outcome data addressed | | | yes | | All participants |  |
|  | free of other bias | | | yes | | The study appears to be free of other sources of bias. |  |
| Exploring Strategies for Attenuating Changes in Bone Mineral Density in MSM on TDF/FTC HIV Pre-exposure Prophylaxis | selection | Is the case definition adequate | | | | yes, with independent validation | A |
|  |  | representativeness of the cases | | | | consecutive or obviously representative series of cases |  |
|  |  | selection of controls | | | | hospital controls |  |
|  |  | definition of controls | | | | no history of disease |  |
|  | comparability | comparability of cases and controls on the bias of the design or analysis | | | | exercise and seasons |  |
|  | exposure | ascertainment | | | | structured interview |  |
|  |  | same method of ascertainment for cases and controls | | | | yes |  |
|  |  | non-response rate | | | | same rate for both group |  |

**Appendix Table 2.** Relationships of Vitamin D supplement and BMD changes

| Studies | Number | Treatment duration（W） | VD intake（IU/D） | Ca intake(mg/D) | BMD change |
| --- | --- | --- | --- | --- | --- |
| Havens, P. L. (2018) | 1 | 48 | 1786 | 0 | 0.25 |
| Overton, E. T. (2015) | 2 | 48 | 4000 | 1000 | 1.86 |
| Boontanondha, P (2020). | 3 | 24 | 2857 | 1250 | 0.1 |
| Nct, (2011) | 4 | 48 | 4000 | 1000 | 1.73 |
| Kortenaar Jean-Luc (2017) | 5 | 48 | 1000 | 1000 | -0.2 |
| Havens, P. L. (2018) | 6 | 48 | 1786 | 0 | 1.06 |
| Overton, E. T. (2015) | 7 | 48 | 4000 | 1000 | 1.71 |
| Boontanondha, P (2020). | 8 | 24 | 2857 | 1250 | 0.4 |
| Pornpaisalsakul, K. (2020) | 9 | 24 | 400 | 1200 | 0.02 |
| Nct, (2011) | 10 | 48 | 4000 | 1000 | 1.5 |
| Thanyawee Puthanakit (2018) | 11 | 24 | 400 | 1500 | 1.15 |
| Kortenaar Jean-Luc (2017) | 12 | 48 | 1000 | 1000 | 0.03 |
